# Mesenchymal stromal cell-derived extracellular vesicles therapy openings new translational challenges in immunomodulating acute liver inflammation

**DOI:** 10.1186/s12967-024-05282-9

**Published:** 2024-05-21

**Authors:** Alexandre Sitbon, Pierre-Romain Delmotte, Valéria Pistorio, Sébastien Halter, Jérémy Gallet, Jérémie Gautheron, Antoine Monsel

**Affiliations:** 1 Multidisciplinary Intensive Care Unit, Department of Anesthesiology and Critical Care, La Pitié-Salpêtrière Hospital, Assistance Publique-Hôpitaux de Paris (APHP), Sorbonne Université, Paris, France; 2 Sorbonne Université, INSERM UMRS-938, Centre de Recherche de Saint-Antoine (CRSA), 75012 Paris, France; 3 Sorbonne Université, INSERM UMRS-959, Immunology-Immunopathology-Immunotherapy (I3), 75013 Paris, France

**Keywords:** Mesenchymal stromal cells, Exosomes, Extracellular vesicles, Acute liver failure, Acute-on-chronic liver failure, Ischemia–reperfusion injury, Immunomodulation, Translational Medicine

## Abstract

Inflammation plays a critical role in conditions such as acute liver failure, acute-on-chronic liver failure, and ischemia–reperfusion-induced liver injury. Various pathogenic pathways contribute to liver inflammation, involving inflammatory polarization of macrophages and Küpffer cells, neutrophil infiltration, dysregulation of T cell subsets, oxidative stress, and activation of hepatic stellate cells. While mesenchymal stromal cells (MSCs) have demonstrated beneficial properties, their clinical translation is limited by their cellular nature. However, MSC-derived extracellular vesicles (MSC-EVs) have emerged as a promising cell-free therapeutic approach for immunomodulation. MSC-EVs naturally mirror their parental cell properties, overcoming the limitations associated with the use of MSCs. In vitro and in vivo preclinical studies have demonstrated that MSC-EVs replicate the beneficial effects of MSCs in liver injury. This includes the reduction of cell death and oxidative stress, improvement of hepatocyte function, induction of immunomodulatory effects, and mitigation of cytokine storm. Nevertheless, MSC-EVs face challenges regarding the necessity of defining consistent isolation methods, optimizing MSCs culture conditions, and establishing quality control measures for EV characterization and functional assessment. By establishing standardized protocols, guidelines, and affordable cost mass production, clinicians and researchers will have a solid foundation to conduct further studies, validate the therapeutic efficacy of MSC-EVs, and ultimately pave the way for their clinical implementation in acute liver injury.

## Background

Cirrhosis stands as a global healthcare challenge, contributing significantly to worldwide mortality and morbidity, ranking as the 11th leading cause of death and the 15th leading cause of morbidity [1]. Characterized by chronic inflammation and tissue fibrosis, it evolves from compensated to decompensated states, with acute-on-chronic liver failure (ACLF) representing the most severe form [2]. Concurrently, acute liver failure (ALF) arises from acute injuries to a previously healthy liver, with multiple etiologies such as viral infections and substance abuse [3]. Both ALF and ACLF involve substantial local and systemic inflammation, leading to multi-organ failure and compromised patient outcomes [3, 4]. In the absence of specific therapies, intensive care and potential liver transplantation (LT) remain the primary treatment modalities [5]. However, LT is fraught with challenges, including organ scarcity, ischemia–reperfusion injury (IRI), and the requirement for lifelong immunosuppression [6]. IRI is a significant mechanism of liver injury that occurs after liver surgeries such as tumor resections, as well as during LT. The generation of reactive oxygen species (ROS) after the restoration of blood flow in ischemic tissue leads to the development of an unfavorable redox state, which plays a critical role in the occurrence of massive cell damage and loss. Therefore, there is an urgent need for novel and effective treatments to improve the survival of patients with ALF or ACLF and to limit IRI during LT [7]. Dysregulated inflammation disrupts liver immune homeostasis, involving a dense population of myeloid and lymphoid immune cells, including Kupffer cells (KCs), paving the way for targeted therapeutic interventions [8]. Mesenchymal stromal cells (MSCs), pluripotent non-hematopoietic stem cells, have shown potential for mitigating liver damage and modulating immune responses [9]. Despite their promise, significant barriers to MSC therapy exist, including uncontrolled differentiation/proliferation and logistical constraints [10]. Currently, it is widely accepted that the immunomodulatory functions of MSCs primarily occur through paracrine mechanisms, largely mediated by the secretion of extracellular vesicles (EVs) with a median size of 100 nm [11]. Mesenchymal stromal cells-derived extracellular vesicles (MSC-EVs) are lipid membrane-bound vesicles capable of mediating intercellular communication by transferring proteins, lipids, and ribonucleic acids (RNAs) between cells [12]. MSC-EVs offer new opportunities in liver injury treatment [7, 13], as they naturally tend to mirror the properties of their parental cell in recapitulating their properties in liver injury, including immunomodulation, anti-fibrotic, anti-apoptotic, and antioxidant effects, while circumventing the pitfalls inherent to the use of MSCs [14]. Upon intravenous administration, MSC-EVs demonstrate a massive accumulation in the liver, which holds particular significance due to the intimate association between liver diseases and imbalances in immune homeostasis [15]. Furthermore, this cell-free based therapy, coupled with the possibility of intravenous route of administration and extended shelf-life and storage capability [16], significantly enhances the translational potential of these therapeutics in human subjects [17].

This review delves into the clinical potential of MSC-EVs in the context of acute liver diseases, focusing on their immunomodulatory effects. It aims to define the molecular mechanisms driving these effects and assess the therapeutic efficacy of MSC-EVs in reducing cell death, improving hepatocyte function, and mitigating cytokine storm. Simultaneously, the review addresses key translational challenges associated with MSC-EVs, including the definition of consistent isolation methods and optimization of culture conditions. The overarching objective is to provide succinct insights into the promising clinical applications of MSC-EVs in acute liver inflammation, acknowledging and navigating through translational obstacles.

### Liver inflammation and immune dysregulation in acute liver injury

ALF and ACLF typically display a rapid and severe onset of systemic and local hyperinflammation, which remains a cardinal feature of their pathophysiology. Triggers such as massive alcohol intake and bacterial infections are responsible for over 96% of ACLF cases [18]. Both ALF and ACLF are characterized with a maladaptive immune response, marked by an overwhelming production of pro-inflammatory cytokines, including tumor necrosis factor (TNF)-α, interleukin (IL)-6, and IL-1β [18].

#### Inflammation and danger molecules-driven signaling pathways

Inflammation is a general response of the immune system to danger signals. Two distinct types of molecular patterns, originating either from the damaged cells of our organism (damage-associated molecular patterns (DAMPs)), or from endogenous or exogenous pathogens (pathogen-associated molecular patterns (PAMPs)), are responsible for local and systemic inflammation. Excessive alcohol intake, for example, can increase gut permeability and induce changes in the composition of the gut microbiome and pH, resulting in increased delivery of endogenous PAMPs—mainly lipopolysaccharide (LPS)—to the liver via the portal circulation, exceeding the clearance capacity of the gut associated lymphoid tissue. On the other hand, in the case of an underlying liver disease, whether it is acute or chronic, hepatocytes and tissue damage result in the release of DAMPs such as adenosine triphosphate (ATP), cholesterol, histones, high mobility group box 1 or desoxyribonucleic acid (DNA) [19].

Circulating DAMPs and PAMPs then bind to pattern recognition receptors such as toll-like receptors (TLRs) and nucleotide-binding oligomerization domain receptors, both of which are upregulated in chronic liver disease. Inflammasomes are intra-cellular pattern recognition receptors mostly sensing injured cells. Inflammasome activation requires two signals: (i) TLR4/LPS-mediated activation prompting nuclear factor-kappa B (NF-κB) activation and nuclear translocation, leading to rapid pro-inflammatory cytokine expression (like TNF-α, monocyte chemoattractant protein-1, IL-6, IL-8, IL-1β)[20]. These cytokines, such as IL-6 and IL-8, are predictors of mortality in ACLF, with higher IL-6 or IL-8 plasmatic concentration when ACLF is precipitated by either bacterial infection or alcohol consumption, respectively [19]. TNF-α is also able to directly activate both apoptotic and necroptotic pathways; (ii) the second signal is typically mediated by DAMPs. Binding DAMPs sensors, such as Nucleotide-binding oligomerization domain, Leucine rich repeat and Pyrin domain containing (NLRPs) or adaptor protein apoptosis-associated Speck like proteins containing a Caspase recruitment domain, triggers inflammasome assembly and activation of pro-caspase-1 to activated caspase-1, leading to IL-1β and IL-18 release[21]. IL-1β, in turn, amplifies inflammation and an extensive array of chemokines production, leading to the recruitment and massive infiltration of neutrophils and pro-inflammatory monocytes/macrophages, as well as activation of resident KCs.

#### Neutrophils and monocytes/macrophages response

In hepatic innate immunity, Küpffer cells (KCs), neutrophils, and monocytes are principal actors. While neutrophils promote phagocytosis, ROS and neutrophil extracellular traps generation, monocytes can differentiate into liver macrophages, accounting for roughly 80% of bodily macrophages. KCs, liver-specific macrophages, are uniquely situated within liver sinusoids for prompt clearance of pathogens and cellular detritus. They regulate hepatic inflammation and participate in tissue repair, thereby maintaining liver immune balance. These cells express TLRs and initiate immune reactions upon sensing PAMPs and DAMPs. In ALF and ACLF, macrophages shift from an anti-inflammatory (M2) to a pro-inflammatory (M1) phenotype, resulting in sustained secretion of cytokines such as TNF-α and IL-6, and generation of ROS and nitric species damaging biological molecules [22]. This imbalance between oxidizing and anti-oxidizing agents, known as oxidative stress (OS), involves hepatocytes, sinusoidal endothelial cells, and KCs in its pathogenesis [23]. Notable features include diminished expression of CXC receptors 1/2 and Nicotinamide-Adenine-Dinucleotide-Phosphate Hydrogen oxidase 2 in neutrophils, and functional changes in monocytes, impairing their phagocytic and ROS-producing capabilities [24]. Additionally, there is an increase in immunosuppressive CD14 + HLA-DR- monocytic cells, undermining antimicrobial defenses [25].

#### Dendritic cells hyperactivation

In the liver, hepatic dendritic cells, originating from CD34 + hematopoietic progenitor cells, are pivotal for modulating both innate and adaptive immune mechanisms. Situated in portal tracts, these antigen-presenting cells mature and migrate to lymphoid organ T-cell regions upon encountering immunogenic stimuli. They activate T helper 17 cells (Th17) and foster regulatory T-cell (Treg) development via IL-10 release. During ALF or ACLF, the multifaceted functions of hepatic dendritic cells appear compromised, resulting in elevated pro-inflammatory activity [26].

#### Lymphocyte imbalance

While the liver is mainly home to innate immune cells, the adaptive immune system plays a key role in the inflammatory dynamics of ALF or ACLF. Research in animal models indicates an imbalanced ratio of Th17 to Treg, characterized by elevated levels of pro-inflammatory cytokines like IL-17A, IL-21, and IL-22 from Th17 cells, as opposed to anti-inflammatory agents like IL-10 and transforming growth factor (TGF)-β from Treg cells [27]. This discord contributes to sustained inflammation. In later stages, adaptive immunity often shows signs of attenuation, reflected by decreased CD4 + and CD8 + T cell populations. Moreover, the suppression of Th17-related cytokines through mechanisms involving Cytotoxic T-lymphocyte-associated protein 4 and HLA-G may usher in an immunosuppressive state [20].

#### Cell death during inflammatory state

Pyroptosis serves as another modality of programmed cell death marked by the expulsion of pro-inflammatory substances, relevant in hepatic inflammation and damage [28]. Regulated through the activation of specific entities like gasdermin D and caspases, primarily caspase-1 and caspase-4/5/11, this process initiates membrane pore formation and eventual cell breakdown [29]. Consequently, intracellular elements, including inflammatory cytokines and DAMPs, are liberated, exacerbating liver inflammation and tissue injury. Various stimuli such as infections, cellular distress, or immunological imbalances can precipitate pyroptosis, which is implicated in liver conditions ranging from inflammation and fibrosis to IRI [29].

#### Reactive hepatic stellate cells activation

Hepatic stellate cells (HSCs), mesenchymal cells similar to fibroblasts and pericytes, play a pivotal role in fibrogenesis [30]. In response to hepatic injury, often mediated by phagocytosis of hepatocyte-derived apoptotic fragments and interactions with KCs, HSCs undergo a transition into myofibroblast-like cells. These activated HSCs, defined by their secretion of extracellular matrix and pro-inflammatory cytokines like IL-1β and IL-18, contribute to liver fibrosis and perpetuate inflammation [31].

#### Contrast in inflammatory responses between acute and chronic liver diseases

Conversely, chronic liver diseases often manifest with a more subdued, albeit persistent, inflammatory state characterized by a gradual shift in macrophage polarization from anti-inflammatory (M2) to pro-inflammatory (M1) phenotypes, contributing to fibrosis and cirrhosis over time. Moreover, chronic conditions involve complex interactions between hepatocytes, HSCs, and KCs, leading to prolonged yet less intense inflammation [20]. Acute liver conditions are marked by a rapid and intense inflammatory response, while chronic diseases display a more protracted and complex inflammatory landscape.

**Fig. 1:**
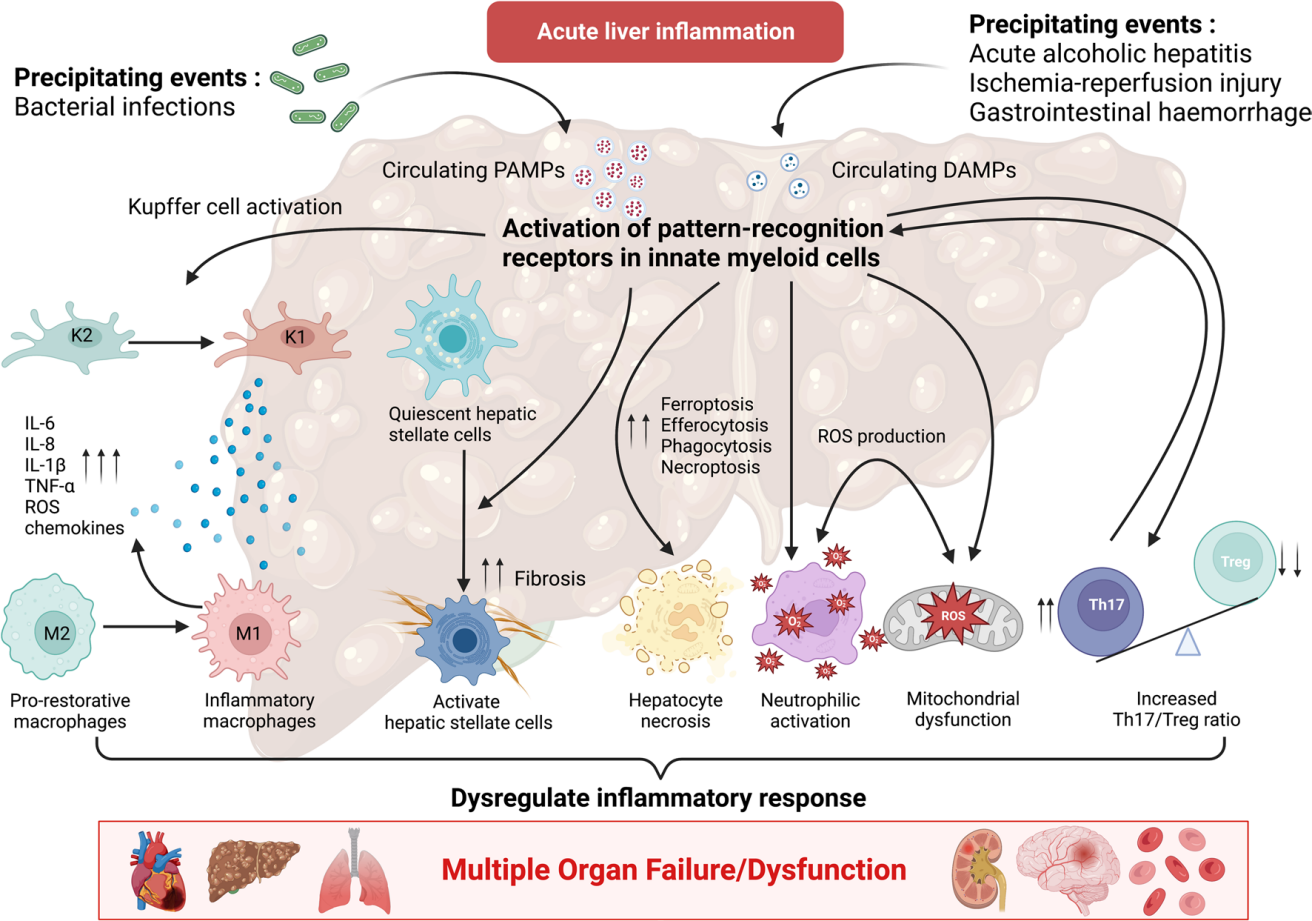
Pathophysiology of acute liver inflammation. Acute liver inflammation is characterized by profoundly dysregulated inflammatory processes, mediated by several effectors, such as M1 macrophage polarization, an increase in the Th17/Treg ratio, hepatic stellate cells activation, hepatocyte necrosis, neutrophil infiltration, and pronounced oxidative stress leading to mitochondrial dysfunction. Il-: Interleukins, TNF: Tumor necrosis factor, ROS: Reactive oxygen species, PAMPs: Pathogen associated molecular patterns, DAMPs: Damage-associated molecular patterns, Th17/Treg: T helper 17 cells/ regulatory T cells. All figures were created with *Biorender.com*

Figure 1 summarizes the mechanisms involved in acute liver inflammation.

### Mechanisms of Immunoregulatory effects of Mesenchymal Stromal Cell-Derived Extracellular Vesicles in Liver Inflammation

#### General mechanisms involved in MSC-EV-based therapies

MSCs are multipotent cells characterized by their spindle-shaped structure, adherence to plastic surfaces, and specific expression of CD markers such as CD73, CD90, and CD105. Originating from diverse tissues like bone marrow and adipose tissue, MSCs can differentiate into multiple cell types like osteoblasts, chondrocytes and adipocytes [32]. Their pleiotropic effects extend to various therapeutic effects in liver diseases, including immunomodulation, anti-fibrotic, and anti-apoptotic activities [33]. While clinical studies support the safety and efficacy of MSCs in treating liver disorders, challenges such as uncontrolled differentiation/proliferation and potential immunogenicity persist [34]. EVs derived from MSCs offer a viable alternative [34]. These nanoscale vesicles, varying in size, enclose a wide array of bioactive molecules, such as micro RNAs, messenger RNAs, and lipids [35]. MSC-EVs recapitulate the effects of MSCs, with crucial advantages such as lower risk of uncontrolled proliferation and immunogenicity, greater stability and much less restrictive storage conditions [36, 37]. MSC-EVs offer a prospective pathway for liver disease treatment, especially given their capacity for long-term storage and clinical convenience. They present a promising avenue for further study into their mechanistic impact on acute liver inflammation and therapeutic applications in liver conditions [13].

#### MSC-EVs in macrophage modulation and liver disease management

Macrophages and KCs significantly influence liver malfunction and related immune response [38]. Multiple studies corroborate the beneficial impact of MSC-EVs on liver functionality, predominantly through macrophage-based anti-inflammatory actions [39]. In ALF, MSC-EVs minimize the release of inflammatory cytokines like IL-6, IL-1β, TNF-α, and IL-17 [40], alongside inflammatory chemokines such as Regulated on Activation, Normal T cell Expressed and Secreted, Monocyte chemoattractant protein-1, and Interferon gamma-induced protein 10 [41]. Conversely, MSC-EVs augment the secretion of anti-inflammatory cytokines like IL-10, IL-1RA, and IL-13 [42], thereby modulating inflammation favorably. Furthermore, they regulate NLRP3 inflammasome activation through micro RNA (miR)-17 and miR-299-3p [43, 44], particularly in the RAW 264.7 macrophage line [45]. Various models of acute autoimmune hepatitis also reported reduced pro-inflammatory cytokine secretion when treated with MSC-EVs. Notably, MSC-EVs modulate Signal transducer and activator of transcription 3 (STAT3) gene expression through miR-223-3p, promoting an anti-inflammatory macrophage phenotype [46]. In IRI post-LT, MSC-EVs led to reductions in systemic inflammation, substantiated by decreased levels of IL-6, IL-1β, and TNF-α [47]. Moreover, a study by Zhou et al. revealed that MSC-EVs with miR-22-3p content can potentiate M2 macrophage polarization in liver transplant settings, exerting strong anti-inflammatory effects [48]. Concerning nonalcoholic steatohepatitis, MSC-EVs play a vital role in ameliorating fibrosis and inflammation. Specifically, they inhibit pro-fibrotic genes like Tissue inhibitor of metalloproteinases-1 and Alpha Smooth Muscle Actin [49], and promote the anti-inflammatory M2 macrophage phenotype, verified by the induction of IL-10 and arginase-1 [50]. In summary, MSC-EVs exhibit promising benefits in mitigating inflammation and fibrosis. By facilitating the polarization of macrophages toward an anti-inflammatory phenotype and suppressing pro-inflammatory mechanisms, MSC-EVs emerge as a viable therapeutic avenue for addressing hepatic disorders.

#### MSC-EVs modulate the immune response by decreasing the ratio of T helper type 17 and regulatory T cells

The imbalance between Th17 and Treg, characterized by an increased Th17/Treg ratio, has been implicated in liver injury and autoimmune diseases [27]. In animal models of ALF and acute autoimmune hepatitis, MSC-EVs have been observed to reduce the Th17/Treg ratio, notably by enhancing the release of miR-223-3p targeting STAT3 pathway, an upstream activator of IL-6 and IL-1β [46]. In liver injury induced by Concavalin A, a notable increase in Treg to CD4 + cell percentages among liver non-parenchymal cells was reported, corresponding to a decline in liver inflammation and necrotic areas [51]. Investigations have also revealed that MSC-EVs attenuate IRI in the liver by adjusting the Th17/Treg balance through miR-1246, impacting the IL-6 signal transducer (gp130) and STAT3 pathway as well as the Wnt/β-catenin pathway [52]. Moreover, they affect the calcium ion influx and regulate the CD154 synthesis in CD4 + T cells, thus modulating their function [53]. The immunomodulatory influence of MSCs on helper T cell subsets in liver, including Th1, Th2, and Th22, has also been established [54]. Similarly, MSC-EVs have exhibited these effects in other organs such as the lungs [55]. A study by Huang et al. demonstrated that MSC-EV-rich conditioned medium altered Th2 cell populations and minimized the Th17/Treg ratio in an ALF mouse model [56]. Conclusively, the ability of MSC-EVs to modulate the Th17/Treg ratio provides a compelling therapeutic avenue for liver disorders, particularly as Tregs are central to immunomoduating mechanisms and tissue repair processes.

#### MSC-EVs could deactivate HSCs, suppress dysregulated cell death (ferroptosis, pyroptosis) and improve liver regeneration

MSC-EVs exhibit therapeutic capabilities in ameliorating toxin-induced liver damage and curtailing HSCs activity [56]. Their efficacy has been documented in preclinical studies of ALF triggered by *S. japonicum*, in which in vitro assays showed MSC-EVs inhibited HSCs proliferation, and in vivo data confirmed enhanced survival rates [57]. In IRI models, MSC-EVs mitigated pyroptotic factors, such as NLRP3 and caspase-1, and enhanced regenerative markers like Cyclin D1 and Vascular Endothelial Growth Factor [58]. A study by Gong et al. supported the involvement of the NF-κB and miR-183/5-lipoxygenase pathways in the mechanism underlying the protective effects of MSC-EVs [47]. MSC-EVs exhibited a protective role against ferroptosis in carbon tetrachloride-induced liver injury by modulating Solute Carrier Family 7 Member 11 function [59]. Moreover, studies have elucidated that MSC-EVs could attenuate ferroptosis by leveraging specific microRNAs and pathways such as Nuclear factor (erythroid-derived 2)-like 2 and miR-124-3p [60, 61]. Thus, MSC-EVs offer a promising avenue for mitigating liver injury, acting through a variety of mechanisms that include HSCs deactivation and regulation of cell death pathways like pyroptosis and ferroptosis.

#### Neutrophil modulation and oxidative stress regulation by MSC-EVs in hepatic injury

MSC-EVs have been demonstrated to be capable of dampening neutrophil-mediated inflammation in liver tissues, largely through mitochondrial transfer to intrahepatic neutrophils, thus aiding in metabolic restoration by modulating the formation of neutrophil extracellular traps [62]. In a rat LT model, MSC-conditioned medium resulted in notable attenuation of neutrophil influx into liver grafts, protecting hepatocytes and sinusoidal endothelial cells [63]. Similarly, the infusion of MSC-EVs in liver IRI models led to diminished inflammatory cytokine levels (IL-6, high mobility group box 1, TNF-α) and reduced OS, accompanied by decreased neutrophil infiltration [64].

#### MSC-EVs in autophagy enhancement and hepatocyte apoptosis attenuation

A seminal study by Lin et al. suggested that MSC-EVs harness the let-7a-5p exosomal component to stimulate autophagy by inhibiting Mitogen-Activated Protein Kinase Kinase Kinase Kinase 3, thereby mitigating inflammation [59]. In the LPS/D-galactosamine mice model, MSC-EVs bolstered autophagy and reduced hepatocyte apoptosis [65]. Furthermore, miR-20a, secreted by MSC-EVs, has been found to regulate apoptosis-related genes, hence lessening hepatocyte apoptosis [66]. Studies have verified that MSC-EVs upregulate pro-survival proteins such as Bcl2 while downregulating pro-apoptotic markers [65]. Two distinct pathways have been identified concerning hepatoprotective effects: one involving Extracellular signal-regulated kinases ½ phosphorylation and Bcl2 overexpression, and the other inhibiting the Inhibitor of nuclear factor kappa B kinase subunit beta/NF-kB/caspase 9/3 pathway [67].

The literature indicates the multifaceted abilities of MSC-EVs in modulating liver injury, from macrophage phenotype shifts to Th17/Tregs balance and OS regulation. MSC-EVs manifest anti-fibrotic, pro-regenerative properties and inhibit stellate cell activation, underscoring their therapeutic potential in liver injury management [68].

Table 1 summarizes preclinical studies on the effects of MSC-EVs in acute liver injury.

**Table 1:** Immunomodulatory influence of mesenchymal stromal cell-derived extracellular vesicles in the context of different acute liver inflammation injury

Disease (model)	Animal/Model	MSCsorigin	Effects	Pathways involved	References	Doi
*Impact on macrophages*						
ALF	Mice/TAA	mBMSC	Promoting macrophage line RAW264.7 apoptosis	NA	*Biao Huang, J Transl Med, 2016*	https://doi.org/10.1186/s12967-016-0792-1
ALF	Mice /LPS-DGalN	mAMSC	Reducing NLRP3 inflammasome activation	↑miR-17; ↓TXNIP/NLRP3	*Yanning Liu, eBioMedicine, 2018*	https://doi.org/10.1016/j.ebiom.2018.08.054
ALF	Mice /LPS-DGalN	hUCMSC	Deceasing expression of NLRP3 and Caspase 1	↓NLRP3; ↓Caspase 1	*Linrui Jiang, Biochem Biophys Res Commun, 2019*	https://doi.org/10.1016/j.bbrc.2018.11.189
ALF	Mice /LPS-DGalN	hUCMSC	Reducing NLRP3 inflammasome activation	↑miR-299-3p; ↓ TXNIP /NLRP3	*Shuquin Zhang, Life Sci, 2020*	https://doi.org/10.1016/j.lfs.2020.117401
ALF	Mice/TAA	hUCMSC	Polarizing M2 Macrophages	NA	*Esteban Fiore, Gene ther, 2020*	https://doi.org/10.1038/s41434-019-0102-7
IRI	Mice/LT	mBMSC	Inducing KCs M2 polarization	↑miR-22-3p; ↓IRF8; ↑M2 and KCs polarization	*Minjie Zhou, J Gene Med, 2023*	https://doi.org/10.1002/jgm.3497
ALF	Mice/Traumatic hemorragic shock	mBMSC	Promoting anti-inflammatory M2 macrophages and KCs	↑PTPN22; ↑IL-10; ↑ CD206	*Yunwei Zhang, Front Immunol, 2021*	https://doi.org/10.3389/fimmu.2021.811164
*Impact on T helper and T reg cells*						
AIH	Mice /ConA	mBMSC	Increasing Treg number	NA	*Ryo Tamura, Inflamm Regen, 2016*	https://doi.org/10.1186/s41232-016-0030-5
IRI	Mice/Left and medial pedicle clamp (90 min) + 6,12, 24 h reperfusion	hUCMSC	Decreasing Th17/treg ratio	↑miR-1246; ↓IL-6; ↓gp30; ↓STAT3	*Kun Xie, IUBMB Life, 2019*	https://doi.org/10.1002/iub.2147
IRI	Mice/Left and medial pedicle clamp (90 min) + 6 h reperfusion	hUCMSC	Dowregulating expression of CD154 synthesis	↑CCT2; ↓CA2 + ; ↓Calcineurin; ↓NFAT1	*Jun Zheng, Adv Sci, 2020*	https://doi.org/10.1002/advs.201903746
AIH	Mice/Hepatic S100 injection	mBMSC	Decreasing Treg/Th17 ratio	↑miR-223-3p; ↓STAT3; ↓IL-1ß; ↓IL-6	*Feng-Bin Lu, Mol Cells, 2019*	https://doi.org/10.14348/molcells.2019.2283
ALF	Mice/TAA	mBMSC	Decreasing Th17/treg ratio	NA	*Biao Huang, J Transl Med, 2016*	https://doi.org/10.1186/s12967-016-0792-1
IRI	Mice/Left and medial pedicle clamp 70% (60 min) + 6 h reperfusion	mBMSC	Upregulation of Treg	NA	*Lei Zheng, Cell Physiol Bioch, 2018*	https://doi.org/10.1159/000488733
*Impact on cell death*						
ALF	Mice /LPS-DGalN	mBMSC	Alleviating hepatocyte ferroptosis	↑p62; ↑Keap1/NRF2	*Shuxian Zhao, Oxid Med Cell Longev, 2022*	https://doi.org/10.1155/2022/8287227
IRI	Rats/Clamping model 70% (80 min) + 24 h reperfusion	rBMSC	Alleviating hepatocyte ferroptosis	↑miR-29a-3p; ↓IREB2, ↓TRF1; ↓Fe2 + ; ↓ferroptosis	*Xiang Li, Oxid Med Cell Longev, 2022*	https://doi.org/10.1155/2022/6520789
ALF	Mice/CCl4	mBMSC	Protecting against ferroptosis	↓Ptgs2/LOXs; ↑SLC7A11; ↑CD44; ↑OTUB1	*Feiyan Lin, Cell Death & Disease, 2022*	https://doi.org/10.1038/s41419-022-04708-w
IRI	Rats/Left and medial clamp + partial hepatectomy + 24 h reperfusion	rAMSC	Inhibiting pyroptosis factors	↓ NF‐kB by:1/ ↑ Wnt/ß-catenin; ↑ Cyclin D1 2/ ↓p65; ↓NLRP3; ↓Caspase1; ↓IL-18	*Chenxi Piao, Int J Mol Sci, 2022*	https://doi.org/10.3390/ijms232012065
IRI	Rats/LT of steatosis donor	rBMSC	Inhibiting ferroptosis	↑miR-124-3p; ↓STEAP3; ↓Fe2 + ; ↓Ferroptosis	*longlong Wu, Journal of Nanobiotechnology, 2022*	https://doi.org/10.1186/s12951-022-01407-8
IRI	Mice/Right pedicle clamp 70% (45 min) + 1–3 days reperfusion	hBMSC	Reducing hepatic necrosis	NA	*Anger, Stem Cell Dev, 2019*	https://doi.org/10.1089/scd.2019.0085
IRI	Pig/Left pedicle clamp (60 min) + 72 h reperfusion	pigAMSC	Reducing apoptosis	↓ NF‐kB	*Cheuk-Kwan Sun, Am J Transl Res, 2017*	PMCID: PMC5411908
IRI	Mice/Left and medial pedicle clamp 70% (60 min) + 1, 3, 6 h reperfusion	mBMSC	Reducing apoptosis	↑CXCL1; ↑F4/80 cells; ↑NLRP12; ↓NF-kB	*Hiroaki Haga, Liver Transpl, 2017*	https://doi.org/10.1002/lt.24770
IRI	Rats/Left and medial pedicle clamp (90 min) + 6 and 24 h reperfusion	hUCMSC	Restoring anormal expression of apoptosis- and autophagy‐related genes	↑miR-20a; ↓Beclin-1 ↓FAS	*Lin Zhang, J Cell Physiol, 2020*	https://doi.org/10.1002/jcp.29264
IRI	Mice/Clamping model 70% (60 min) + 3 h reperfusion	hUCMSC	Reducing apoptosis	↑MALAT1	*Maryam Sameri, Biochem Biophys Res Commun, 2022*	https://doi.org/10.1016/j.bbrc.2022.09.111
*Impact on neutrophil modulation and oxidative stress disruption*						
IRI	Rats/LT of steatosis donor	rBMSC	Inhibiting neutrophil infiltration	↑miR-124-3p	*longlong Wu, Journal of Nanobiotechnology, 2022*	https://doi.org/10.1186/s12951-022-01407-8
IRI	Mice/Clamping model 70% (90 min) + 6 h reperfusion	hUCMSC	Reducing neutrophil extracellular traps formation	NA	*Tongyu Lu, Biomaterials, 2022*	https://doi.org/10.1016/j.biomaterials.2022.121486
IRI	Rats /Left and medial pedicle clamp (90 min) + 6 and 24 h reperfusion	hUCMSC	Reducing neutrophil infiltration, alleviating oxidative burst	↑MnSOD	*Jia Yao, FASEB, 2019*	https://doi.org/10.1096/fj.201800131RR
IRI	Rats/Left and medial clamp 70% (60 min) + 2 and 6 h reperfusion	rAMSC	Providing strong antioxidative effects	PGE2 pathway: 1/ ↑ERK1/2; ↓ Bax 2/ ↑GSK-3β; ↑Bcl-2,β -catenin	*Yaqing Zhang, Int J Mol Med, 2022*	https://doi.org/10.3892/ijmm.2021.5068
ALF	Mice /CCl4	hUCMSC	Inducing strong antioxidative effect	1/↑ERK 1/2; ↑Bcl2/p65 2/↓ IKKB; ↓NF‐kB; ↓caspase 9/3	*Yongmin Yan, Mol Ther, 2017*	https://doi.org/10.1016/j.ymthe.2016.11.019
IRI	Rats /Portal and artery clamp (30 min) + Partial hepatectomy	rBMSC	Inducing antioxidative and antiapoptotic effects	NA	*Apeksha Damania, Stem Cell Res Ther, 2018*	https://doi.org/10.1186/s13287-017-0752-6
ALF	Rats/LPS-DGalN	rBMSC	Decreasing oxidative stress	NA	*Li Chen, Stem Cell Int, 2018*	https://doi.org/10.1155/2018/9156560
IRI	Rats/Left and medial pedicle clamp 70% (30 min) + left hepatectomy + 24 h reperfusion	rAMSC	Reducing liver levels of total oxidant status	↑Bcl2/p65 ↓Bax; ↓caspase 9/3	*Qianzhen Zhang, J Cell Mol Med, 2021*	https://doi.org/10.1111/jcmm.16952

In preclinical investigations, mesenchymal stromal cells-derived extracellular vesicles (MSC-EVs) have exhibited multiple functionalities that enhance hepatic function and augment survival rates following episodes of acute liver inflammation. Specifically, MSC-EVs have been observed to mitigate the severe systemic inflammatory response while concurrently exerting robust immunomodulatory influences. These are achieved through the regulation of macrophage and Kupffer cells activity, correction of Th17/Treg imbalances, attenuation of oxidative stress, reduction in neutrophil infiltration, and modulation of cellular apoptosis. The accompanying table elucidates the specific effects and pathways implicated in the role of MSC-EVs in acute hepatic inflammation

AIH: Acute immune hepatitis; ALF: Acute liver failure; AMSC: adipose tissue-derived mesenchymal stromal cells; BMSC: bone marrow-derived mesenchymal stromal cells; CCL_4_: Carbon Tetrachloride; CCT2: Chaperonin Containing TCP1 Subunit 2; CD-: cluster differentiation; ConA: Concavalin A; CXCL1: stromal cell-derived factor 1; DGalN: D-galactosamine; ERK: Extracellular signal-regulated kinases; EVs: Extracellular vesicles; h-/m-/r-MSC: human-MSC/mice-MSC/rat-MSC origin; GSK: glycogen synthase kinase; IKKB: Inhibitor Of Nuclear Factor Kappa B Kinase; IL-: Interleukine-; IREB2: iron responsive element binding protein 2; IRF8: Interferon regulatory factor 8; IRI: ischemia–reperfusion injury; KCs: Küpffer cells; LPS: Lipopolysaccharide; LT: Liver transplantation; MALAT1: metastasis lung adenocarcinoma transcript 1; MnSOD: manganese-dependent superoxide dismutase; NFAT: Nuclear factor of activated T-cells; NF‐kB: nuclear factor-kappa B; NLRP3: NOD-like receptor family, pyrin domain containing; NRF2: Nuclear factor (erythroid-derived 2)-like 2; PTPN22: Protein tyrosine phosphatase, non receptor type 22; STAT3: Signal transducer and activator of transcription,3; STEAP3: Six-Transmembrane Epithelial Antigen of Prostate 3; TAA: Thioacetamide; Th: T helper cells, Treg: regulatory T cells; TRF1: Telomeric Repeat Factor 1; TXNIP: Thioredoxin Interacting Protein; UCMSC: umbilical cord-derived mesenchymal stromal cells

**Fig. 2:**
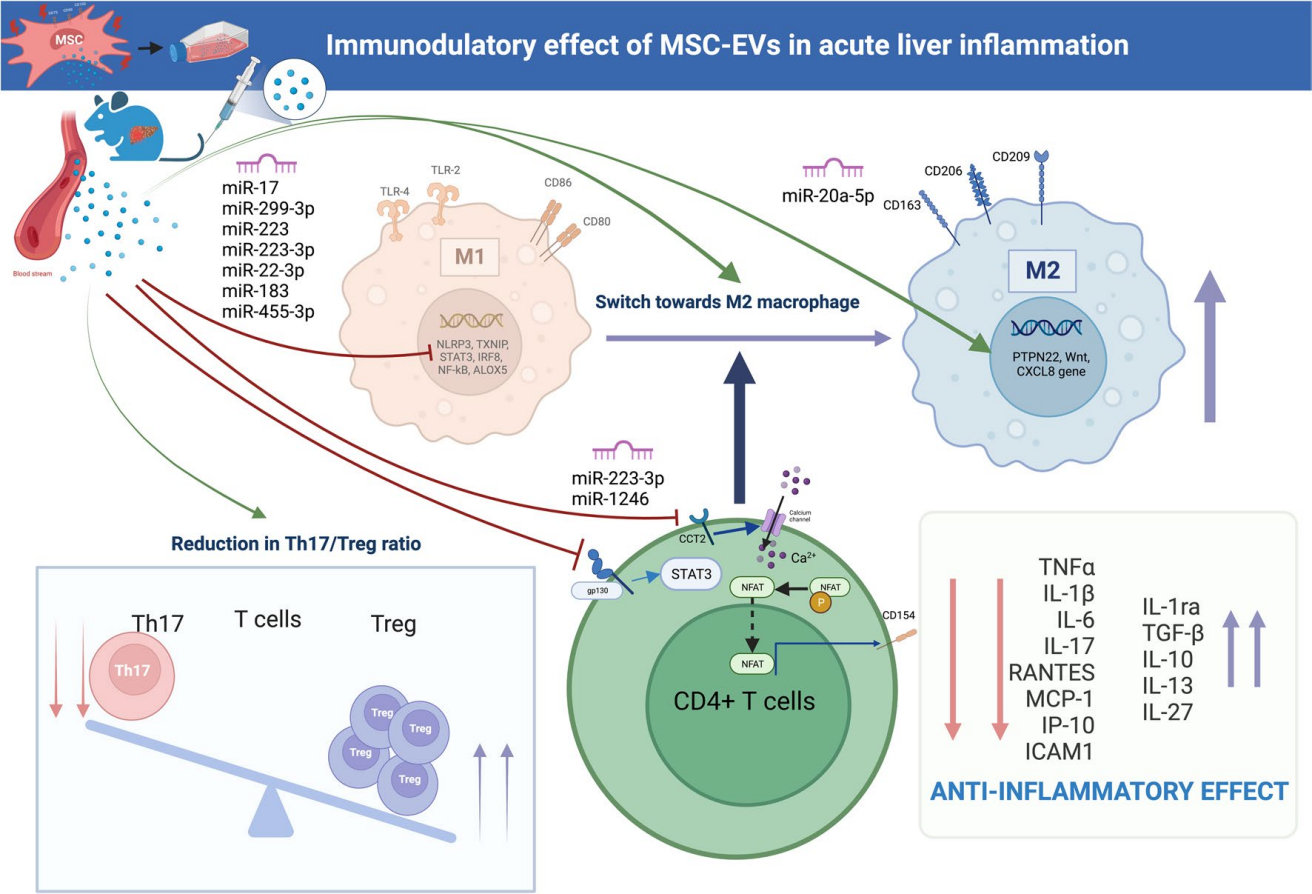
Immunomodulatory effects of MSC-EVs in acute liver inflammation, focusing on macrophages and CD4 + T cells effects. Macrophages and CD4 + T cells play a pivotal role in the immunomodulatory effects of MSC-EVs in acute liver inflammation. Through the transduction of several miRNAs that target specific genes involved in the inflammatory process, MSC-EVs demonstrate a remarkable ability to attenuate acute liver inflammation. Consequently, the influence of MSC-EVs on both local and systemic hepatic inflammation leads to a decrease in the Th17/Treg ratio, polarization of macrophages and Kupffer cells (KCs) towards an anti-inflammatory phenotype, and immunomodulation of cytokine and chemokine secretion. MSCs: Mesenchymal stromal cells, MSC-EVs: Mesenchymal stromal cells-derived extracellular vesicles, miR: micro-RNA, Il-: Interleukines, TNF: Tumor necrosis factor, Th17/Treg: T helper 17 cells/ regulatory T cells, CD: Cluster of differentiation, TLR: Toll-like receptor, NLRP3: NOD-like receptor family, pyrin domain containing 3, TXNIP: Thioredoxin Interacting Protein, STAT3: Signal transducer and activator of transcription 3, IRF8: Interferon regulatory factor 8, NF-κB: nuclear factor-kappa B, ALOX5: Arachidonate 5-Lipoxygenase, CCT2: Chaperonin Containing TCP1 Subunit 2, NFAT: Nuclear factor of activated T-cells, RANTES: Regulated on activation, normal T cell expressed and secreted, MCP-1: Monocyte chemoattractant protein-1, IP-10: Interferon gamma-induced protein 10, ICAM1: InterCellular adhesion molecule, TGF: transforming growth factor, PTPN22: rotein tyrosine phosphatase non-receptor type 22. All figures were created with *Biorender.com*

The Figure 2 provides a comprehensive summary of the molecular effects of MSC-EVs in the context of acute liver inflammation, with a specific emphasis on the influence exerted on macrophages and CD4 + T cells.

### Translational challenges of mesenchymal stem/stromal cells-derived extracellular vesicles in acute liver inflammation

Despite the promise MSC-EVs hold as a cell-free therapy, various complexities and inconsistencies impede their translational journey to clinical settings [17]. Contributing to these limitations are variances in MSCs source tissues (whether bone marrow, adipose deposits, or human umbilical cords) as well as inconsistencies in the therapeutic efficacy across different production batches. The challenge extends to establishing the ideal dosage and treatment regimen, and determining the most effective route of administration for MSC-EVs. [69]. Additional layers of complexity arise from heterogeneous culture conditions, including hypoxic environments or conditions enhanced by growth factors, and the challenge of scaling up the mass production process in a standardized approach. Regulatory and technical intricacies, unique to MSC-EVs, further restrict research access to clinical applications, thus delaying the assessment of MSC-EVs as emergent therapeutic solutions.

#### Definition and characterization of MSC-EVs

Divergent views and debates persist concerning the biological attributes, functional roles, and potency assays pertinent to MSC-EVs, complicating efforts for uniform definitions and characterizations [35]. The identification of MSCs is principally rooted in the characterization of their progenitor cells and their originating tissue. Factors such as donor age, passage history of the MSCs, and variations between donors further add layers of complexity to MSCs characterization. Protocols for isolating MSCs, coupled with diversity in culture conditions (*e.g.*, 2D versus 3D culture), also influence their features. Additionally, MSC-EVs profiling incorporates not only the traits of the MSCs but also the criteria set forth by the Minimal Information for Studies of Extracellular Vesicles 2018 guidelines. These standards address aspects such as the dimensional properties of EVs and surface molecule expression [13]. Although strides have been made by the International Society for Cell and Gene Therapy to bring uniformity to characterization techniques, inconsistencies remain. Diverse methods, including nanoparticle tracking, protein and mRNA assessments, electron microscopy, western blotting, and flow cytometry, contribute to the heterogeneity [35]. The absence of universally accepted potency assays for confirming MSC-EVs efficacy in specific pathologies complicates matters further. Such variability poses challenges to the consistency of therapeutic outcomes and comparability across studies [70]. In terms of tissue source selection for parental MSCs and MSC-EVs quantification and quality assessment, there is a pressing need for standardization. Additionally, donor selection demands caution given the risk of viral transmission and variable MSCs quality from different patients. The overarching goal is to correlate particular features of each MSC-EVs batch with their inherent biological traits and therapeutic efficacy [71].

#### Clinical applications

Defining the standardized therapeutic dosage for MSC-EVs represents a significant obstacle. Existing literature commonly prescribes higher concentrations of MSC-EVs relative to MSCs, complicating matters further [72]. In animal models, reported therapeutic dosages span a broad range from 0.1 to 250 μg of EV protein content, or between 2.0 × 10^8^ and 5.0 × 10^11^ particles per dose, assuming 1 μg of EV protein content is approximately equivalent to 2.0 × 10^9^ MSC-EVs [73]. Optimal routes for MSC-EVs administration continue to be a topic of research. Although MSC-EVs have a brief plasma half-life, significant concentrations have been found in the liver 24 h post-infusion [74]. A recent study revealed that intravenous administration led to noticeable accumulations of MSC-EVs in the liver and spleen, starting from 3 h post-infusion and lasting for 24 h [15]. Conversely, intratracheal and intranasal routes failed to result in localized accumulations, suggesting intravenous methods may be preferable for liver-targeted therapies in murine models [15]. The best route for human applications (whether intravenous, intrasplenic, portal, or intra-arterial) remains to be explored. Another area requiring clarification is the efficacy of single *versus* multiple injections, particularly within the context of MSC-EVs therapeutic applications. Determining an optimal treatment schedule is crucial for maximizing patient outcomes. Notably, clinical investigations have so far indicated a favorable safety profile for MSC-EVs, even during the COVID-19 pandemic when they were administered to critically-ill patients through nebulized [75] or intravenous routes [76], without any significant adverse events. Nevertheless, more exhaustive long-term safety studies are essential to further substantiate the safety of this cell-free therapy.

#### Scaling-up, isolation, and bioengineering of clinical-grade MSC-EVs: challenges and innovations

One pressing issue in the production of clinical-grade MSC-EVs is the necessity to scale-up MSC production, a process resource-intensive and dependent on the accessibility of expansive culture apparatus [77]. Innovations like 2D and 3D bioreactors are emerging to facilitate large-scale MSC-EVs production. However, considerable variability exists in the methodologies, from expansion methods such as 2D vs 3D culture systems, to various stress conditions like hypoxia or serum starvation imposed on MSCs [17]. Currently, three cutting-edge techniques are employed in the bioproduction of 3D-cultured MSCs: bioreactor-based 3D culture of MSCs, recellularization of MSCs using a 3D scaffold, and the 3D printing of these scaffolds [78]. For instance, the impact of a 3D-printed scaffold-perfusion bioreactor system on the production and bioactivity of EVs secreted by MSCs has been explored. Findings suggest that culture in a perfusion bioreactor can yield an approximate 40–80-fold increase in MSC EVs production, varying with the measurement technique, compared to traditional cell culture methods [79]. Although the application of MSC EVs produced via this system in liver disease has not yet been fully evaluated, the approach holds substantial potential for future therapeutic interventions. The isolation of MSC-EVs represents another hurdle, with techniques ranging from differential ultracentrifugation to tangential flow filtration and size exclusion chromatography [80]. TFF is increasingly recognized as the most efficient for scaling, overcoming limitations associated with other techniques like ultracentrifugation. Standardized isolation protocols are urgently needed to guarantee product consistency. Storage and preservation of MSC-EVs offer their own set of challenges, although evidence supports their stability at − 80 °C for up to 6 months [37]. Short-term storage stability has also been indicated between temperatures of 0–4 °C for up to 8 days [81]. Various formulations and buffer solutions, such as PBS-human albumin trehalose, can significantly enhance MSC-EVs stability during multiple freeze–thaw cycles and long-term storage [81]. Thus, MSC-EVs present a safer, more versatile option for therapeutic use compared to MSCs. They offer a "cell-free" therapy without nuclei, reducing the risks of uncontrolled proliferation and immunogenicity, and preventing the potential side effects associated with MSCs, such as pulmonary capillary overload or occlusion. Their nanovesicle structure enhances safety, particularly during intravenous administration [7]. MSC-EVs are stable, easy to store, and can be used as ready-to-use medicinal products, withstanding long-term storage and freeze–thaw cycles better than MSCs. Clinically, they have a strong safety profile, proven in various trials including COVID-19 treatments [82]. Advances in bioengineering enable MSC-EVs to be customized with specific molecules, making them a targeted therapeutic option. These combined factors highlight MSC-EVs' efficacy and safety, enhancing their clinical appeal [7]. Advancements in bioengineering open new vistas for MSC-EVs as drug delivery vehicles, potentially customizable for tissue targeting or enhanced blood stability [83]. Leveraging the biogenesis of EVs for bioengineering and therapeutic cargo loading represents a novel application of MSC-EVs in liver disease treatment. Future developments will focus on engineering MSC-EVs to contain specific microRNAs or proteins derived from genetically modified MSCs. Another process involves endogenous engineering approaches, such as luminal or surface modifications in MSC-EVs. Additionally, direct loading of nucleic acids into EVs with targeted microRNAs or proteins is possible, further enhancing their therapeutic potential [84]. Bio-engineered MSC-EVs can potentially overcome physiological barriers such as the blood–brain barrier, expanding their range of clinical application [85].

Future studies will need to determine the therapeutic benefits of these modifications, thereby warranting standardized procedures based on clinical objectives and targeted pathologies.

#### Quality control and regulatory aspect

Certainly, to assure consistent quality of MSC-EVs in both regular manufacturing and product surveillance, identification of critical quality attributes is crucial. Rohde et al. have suggested a multi-faceted approach to quality control, incorporating evaluations of progenitor cell properties, EV characteristics, microbial purity, and functional potency [12]. These assessments provide a comprehensive view of the quality and therapeutic effectiveness of MSC-EVs. Moreover, employing a reference product can standardize batch-to-batch comparisons, serving as a quality benchmark and aiding in the detection of any notable disparities [80]. These rigorous quality controls facilitate continuous oversight throughout MSC-EVs production, ensuring that the end product is up to defined specifications and norms [86]. Despite the advantages and potential for easier and faster regulatory approval of MSC-EVs therapies compared to cell-based treatments, several challenges remain in their clinical translation. In terms of regulatory rules, EVs are subject to different rules from those established for cell-based therapies, and recent papers have provided important guidance on the regulatory aspects of their pharmaceutical development. Several initiatives from the International Society for Extracellular Vesicles Task Force on Regulatory Affairs and Clinical Use of EV-based Therapeutics [87] as well as the Exosomes Committee from the ISCT, as well as the French work group “Extracellular Vesicle translatiOn to clinicaL perspectiVEs - EVOLVE France” [13], are working on these aspects and have already published several guidelines enabling investigators to classify their EV therapeutics pragmatically so as to be able to compose their investigational medicinal product dossier in order to advance in clinical translation. Overall, the categorization of EV-based therapies will depend on the cell type of origin, whether the final EVs are native or modified, their formulation, and their mode of administration [13, 87]. While regulatory frameworks may differ between continents, the European framework allows EV-based therapeutic products, under development or to be developed, to be included in the definition of medicinal product under Directive 2001/83/EC. Within the medicinal products framework, EV-based products are categorized as “biological medicinal products” (Directive 2003/63/EC). The subcategorization of EV-derived products will take into account their complexity and active substance, as proposed in an ISEV position paper [87]. Thus, the international societies stresses the need for stringent validation processes for any interventions designed to alter EV content and underscores the priority of establishing the safety profile of MSC-EVs-based therapy in initial clinical trials.

## Conclusion and perspectives

This comprehensive review explored the pivotal role of dysregulated inflammation in the pathogenesis of both ALF and ACLF, conditions characterized by significant clinical severity. Notably, MSC-EVs can tilt the balance of polarization towards type-2 regulatory macrophages, regulate Th17/Treg ratios, reduce neutrophil infiltration, and alleviate OS. Additionally, they modulate hepatocyte autophagy and apoptosis while inhibiting HSC activation. The cumulative impact of these immunomodulatory, anti-inflammatory, and regenerative effects underscores the emerging potential of MSC-EVs as a groundbreaking therapeutic intervention for the management of ALF and ACLF. Nevertheless, the feasibility of their clinical translation and the therapeutic reliability of MSC-EVs are highly dependent on rigorous standardization of manufacturing protocols. This necessitates the development of uniform isolation techniques, optimization of MSCs culture conditions, and the implementation of rigorous quality control metrics for both EVs characterization and functional evaluation. Through the establishment of such standardized procedures, comprehensive guidelines, and economically viable mass production, the scientific community is poised to conduct robust investigative studies. This will not only facilitate the validation of MSC-EVs’ therapeutic utility but also catalyze their clinical adoption for the effective management of liver injuries. As the field advances, continued research endeavors and collaborative efforts are imperative to unlock the full therapeutic potential of MSC-EVs in the complex landscape of liver diseases.

## Materials and methods

### Eligibility criteria

We performed a scoping review by selecting the most relevant articles by combining “Mesenchymal stromal cells”, “Mesenchymal stem cells”, “extracellular vesicles”, “exosomes”, “acute liver failure”, “acute-on-chronic liver failure”, “ischemia–reperfusion injury”, “immune regulation”, “liver immunity”, “immunomodulation”, “inflammation”, “Macrophage polarization”. We have not taken any restrictions regarding the date or location of the publications. The final search occurred on First July of 2023. We reviewed our findings to identify work relevant to immunomodulatory effect of MSC-EVs in liver injury.

### Information sources

Our research is conducted in several databases such as PubMed, Web of Science, Cross Ref, and Google scholar. This review was writing in accordance with the PRISMA statement.

## Data Availability

Not applicable.

## Abbreviations

ALF: Acute liver failure
ACLF: Acute-on-chronic liver failure
ATP: Adenosine triphosphate
DAMPs: Danger/Damage-associated molecular patterns
DNA: Desoxyribonucleic acid
EVs: Extracellular vesicles
HSCs: Hepatic stellate cells
IL-: Interleukin-
IRI: Ischemia-reperfusion injury
KCs: Küpffer cells
LPS: Lipopolysaccharide
LT: Liver transplantation
miR: Micro RNA
MSC-EVs: Mesenchymal stromal cells-derived extracellular vesicles
MSCs: Mesenchymal stromal cells
Nf-κb: Nuclear factor-kappa B
NLRP: Nucleotide-binding oligomerization domain, Leucine rich repeat and Pyrin domain containing
OS: Oxidative stress
PAMPs: Pathogen-associated molecular patterns
ROS: Reactive oxygen species
RNAs: Ribonucleic acids
STAT3: Signal transducer and activator of transcription 3
Th: T helper cells
TLRs: Toll-like receptors
TNF: Tumor necrosis factor
Treg: Regulatory T cells
